# Cerebellar lobular morphometry in trigeminal neuralgia: cross-sectional and exploratory postoperative analyses

**DOI:** 10.3389/fneur.2026.1964745

**Published:** 2026-10-22

**Authors:** Huaiyi Li, Jinying Chen, Xiaoqian Wang, Jingjing She, Weiwei Gao, Tao Wu

**Affiliations:** 1 Department of Radiology, The Second Affiliated Hospital of Xiamen Medical College, Xiamen, China; 2 Department of Neurology, Zhongshan Hospital of Xiamen University, Xiamen, China; 3 Department of Cardiology, Xiamen Humanity Hospital, Fujian Medical University, Xiamen, Fujian, China; 4 Department of Neurology, The First Affiliated Hospital of Hunan University of Medicine, Huaihua, China; 5 Department of Radiology, The First Affiliated Hospital of Hunan University of Medicine, Huaihua, China

**Keywords:** cerebellar lobules, cortical thickness, DeepCERES, longitudinal imaging, morphometry, trigeminal neuralgia

## Abstract

**Background:**

Cerebellar morphometric findings in trigeminal neuralgia (TN) are inconsistent, and lobular volume and cortical thickness are poorly characterized. We compared cerebellar lobular morphometry between patients with TN and healthy controls (HCs) and explored postoperative changes.

**Methods:**

We included 86 patients with TN and 40 HCs; 49 patients had paired preoperative and postoperative magnetic resonance imaging (MRI). DeepCERES was used to quantify bilateral volume and MRI-derived mean cortical thickness in 12 cerebellar regions. Cross-sectional regression models adjusted for age (linear and quadratic terms) and sex; volume models additionally adjusted for intracranial volume (ICV). Longitudinal changes were assessed using paired *t* tests. False discovery rate (FDR) correction was applied separately to the 12 tests for each measure.

**Results:**

No regional or global cerebellar volume differences were significant after FDR correction in the cross-sectional analysis. Patients with TN had higher MRI-derived mean cortical thickness in Lobules IV and VI than HCs. Adjusted differences were 0.104 mm (95% confidence interval [CI], 0.040–0.168; *q* = 0.020) for Lobule IV and 0.071 mm (95% CI, 0.021–0.121; *q* = 0.036) for Lobule VI. The Lobule IV difference was significant across the age models examined, whereas the Lobule VI finding was sensitive to how age was modeled. Among the 49 patients with paired MRI, volume increased postoperatively in Lobules IV–VI, VIIb, VIIIa, and VIIIb, and thickness increased in Lobules IV–VI (all *q* < 0.05). Across these regions, the mean of individual percentage changes ranged from 0.86 to 1.69% for volume and from 1.38 to 3.00% for thickness. Three of the six volume increases and all three thickness increases remained significant after FDR correction in sensitivity models accounting for changes in ICV and automated quality scores.

**Conclusion:**

Patients with TN had higher MRI-derived cortical thickness in Lobules IV and VI than HCs, with the most consistent evidence in Lobule IV. No significant between-group differences in cerebellar volume were detected. Postoperative increases in volume and thickness across several lobules are exploratory findings whose biological significance requires further investigation.

## Introduction

Trigeminal neuralgia (TN) is a craniofacial neuropathic pain disorder characterized by recurrent, brief episodes of severe, electric shock-like pain in the distribution of one or more divisions of the trigeminal nerve (1, 2). Classical TN is associated with neurovascular compression (NVC) at the trigeminal nerve root entry zone and morphological changes in the nerve root. However, the degree of NVC on imaging does not consistently correspond to pain intensity, attack characteristics, or treatment outcomes (1, 2), suggesting that local NVC alone may not account for the clinical heterogeneity of TN. Multimodal magnetic resonance imaging (MRI) studies have reported abnormalities in gray matter morphology, white matter microstructure, and functional connectivity involving the thalamus, insula, cingulate cortex, basal ganglia, sensorimotor cortex, and prefrontal cortex in patients with TN (3–8). The affected regions and direction of change vary considerably across studies (9–11), and cerebellar findings are particularly inconsistent. Some single-center studies have reported reduced cerebellar gray matter volume or other cerebellar structural abnormalities (3–5, 12). A meta-analysis that included both TN and trigeminal neuropathic pain also found lower gray matter density in the anterior cerebellar lobe (13). In contrast, meta-analyses restricted to TN have not identified consistent cerebellar structural abnormalities across studies (9–11). It therefore remains unclear whether patients with TN have cerebellar morphometric differences and, if so, how these differences are distributed.

Different cerebellar regions serve distinct functions, and global volume measurements may obscure regional morphometric differences. The anterior lobe is primarily involved in sensorimotor and somatosensory processing, whereas posterior regions such as Crus I and Crus II contribute more to cognitive and affective processes (14, 15). Studies in healthy volunteers have shown that nociceptive trigeminal stimulation activates multiple cerebellar lobules. Stimulation of territories innervated by different trigeminal divisions and the occipital nerve also elicits somatotopically organized cerebellar responses (16, 17). These findings provide a functional anatomical rationale for examining TN-related morphometry at the lobular level. Most previous morphometric studies in TN have used whole-brain voxel-based morphometry (VBM) or surface-based analyses of the cerebral cortex. Few studies have specifically quantified cerebellar lobules, and cortical thickness studies have also focused mainly on the cerebral cortex (7, 8). CERES and DeepCERES automatically segment cerebellar lobules and provide estimates of lobular volume and mean cortical thickness from MRI (18, 19), enabling the two measures to be examined together.

We used DeepCERES to quantify bilateral volume and MRI-derived mean cortical thickness in 12 cerebellar regions and compared patients with TN with healthy controls (HCs). We aimed to characterize the regional distribution of morphometric differences and assess whether volume and thickness findings were consistent. Within the TN group, we examined associations with disease duration, Sindou grade, and pain side, and explored cerebellar asymmetry in relation to pain side. We also conducted an exploratory longitudinal analysis in patients with paired preoperative and postoperative MRI to describe postoperative changes in cerebellar morphometry.

## Materials and methods

### Study design and participants

We conducted a secondary analysis of the OpenNeuro ds005713 dataset, comprising baseline cross-sectional group comparisons and an exploratory longitudinal analysis of paired preoperative and postoperative MRI (20–22). We combined dataset versions 1.0.1 and 2.0.2 and removed duplicate records by participant identifier. The original study was approved by the Ethics Committee of the Federal Neurosurgical Center and conducted in accordance with the Declaration of Helsinki. All participants provided written informed consent and agreed to the public sharing of deidentified data.

We excluded participants without usable baseline MRI or essential demographic information (age and sex), those with coexisting trigeminal autonomic cephalalgias or previous TN-related surgery, and those with structural, vascular, or space-occupying intracranial abnormalities that could affect cerebellar segmentation or morphometric measurements. Participants with Fazekas grade 3 white matter lesions were also excluded. Exclusions among HCs included intracranial mass lesions, idiopathic intracranial hypertension, a persistent trigeminal artery, and asymptomatic NVC documented in imaging reports. The final baseline sample comprised 86 patients with TN and 40 HCs. Of the patients with TN, 49 had usable paired preoperative and postoperative MRI. Longitudinal inclusion was based on the availability of paired images; complete postoperative clinical records and follow-up timing records were not required. The selection process is shown in Figure 1 and Supplementary Table S1. Detailed eligibility criteria and reasons for exclusion are provided in the Supplementary material.

**Figure 1:**
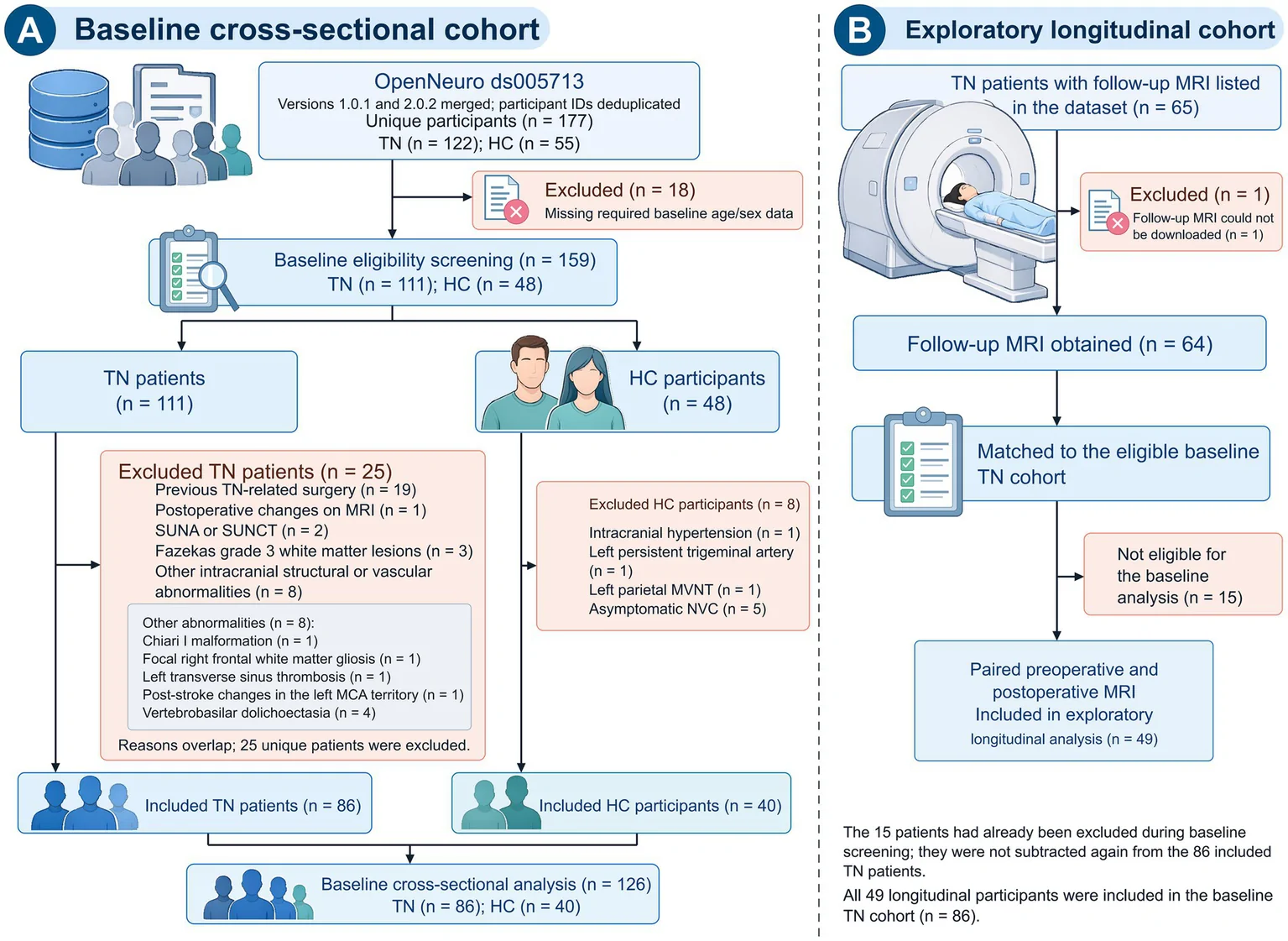
Participant selection for the cross-sectional and exploratory longitudinal analyses. **(A)** Dataset versions 1.0.1 and 2.0.2 of OpenNeuro ds005713 were combined and deduplicated by participant identifier, yielding 177 unique participants. After excluding 18 participants without the required baseline age or sex information, 159 underwent eligibility screening. The final cross-sectional sample comprised 86 patients with trigeminal neuralgia (TN) and 40 healthy controls (HCs). TN exclusion reasons overlapped; the reported categories correspond to 25 unique excluded patients. **(B)** Follow-up MRI was listed for 65 patients and obtained for 64. Of these, 15 did not meet baseline eligibility criteria, leaving 49 patients with paired preoperative and postoperative MRI. These 49 patients were a subset of the 86 baseline TN patients; the 15 ineligible patients were not subtracted again from that cohort. MRI, magnetic resonance imaging; SUNA, short-lasting unilateral neuralgiform headache attacks with cranial autonomic symptoms; SUNCT, short-lasting unilateral neuralgiform headache attacks with conjunctival injection and tearing; NVC, neurovascular compression; MVNT, multinodular and vacuolating neuronal tumor; MCA, middle cerebral artery.

### Clinical and neuroimaging assessments

We extracted age, sex, and the main MRI findings for all participants, together with pain side, pain phenotype, disease duration, medication records, and Sindou grade for patients with TN. Sindou grades were taken from the original neuroradiologist’s imaging assessment of neurovascular relationships at the cerebellopontine angle. For the longitudinal subgroup, surgical procedures were obtained from all_data.csv. Available clinical information was extracted from participants.tsv and included the average pain score during the week before admission, percentage pain reduction immediately after surgery, numerical rating scale (NRS; 0–10) pain score at the second telephone follow-up, and days from surgery to clinical follow-up. Postoperative clinical data were summarized only for patients with the relevant records. Clinical variable definitions, the sequence of assessments, and the extent of missing data are described in the Supplementary material.

### Magnetic resonance imaging acquisition

All MRI scans were acquired on a 3.0-T Philips Ingenia scanner (Philips Healthcare, the Netherlands) with a 16-channel head coil. High-resolution sagittal three-dimensional T1-weighted images were acquired using a fast field echo sequence with a repetition time of approximately 6.6 ms, an echo time of approximately 3.0 ms, and a flip angle of 8°. The acquired voxel size was 1.0 mm isotropic, and images were reconstructed by interpolation to approximately 0.5 mm isotropic resolution. When an examination included multiple T1-weighted acquisitions, the first acquisition was used for morphometric analysis.

### Cerebellar lobular segmentation and morphometric assessment

All included T1-weighted images were processed using DeepCERES v1.1 on the volBrain platform (19) (Figure 2). Preoperative and postoperative images were processed independently, without a subject-specific longitudinal pipeline that jointly processed the two scans. The main DeepCERES processing steps include denoising, affine registration to Montreal Neurological Institute 152 (MNI152) standard space, N4 bias field correction, intensity normalization, and intracranial cavity segmentation. The pipeline then uses super-resolution to reconstruct T1-weighted images at 0.5 mm isotropic resolution and generates synthetic T2-weighted images and subject-specific multi-atlas priors. These inputs are passed to an ensemble segmentation model to produce cerebellar gray matter, white matter, and bilateral lobular labels.

**Figure 2:**
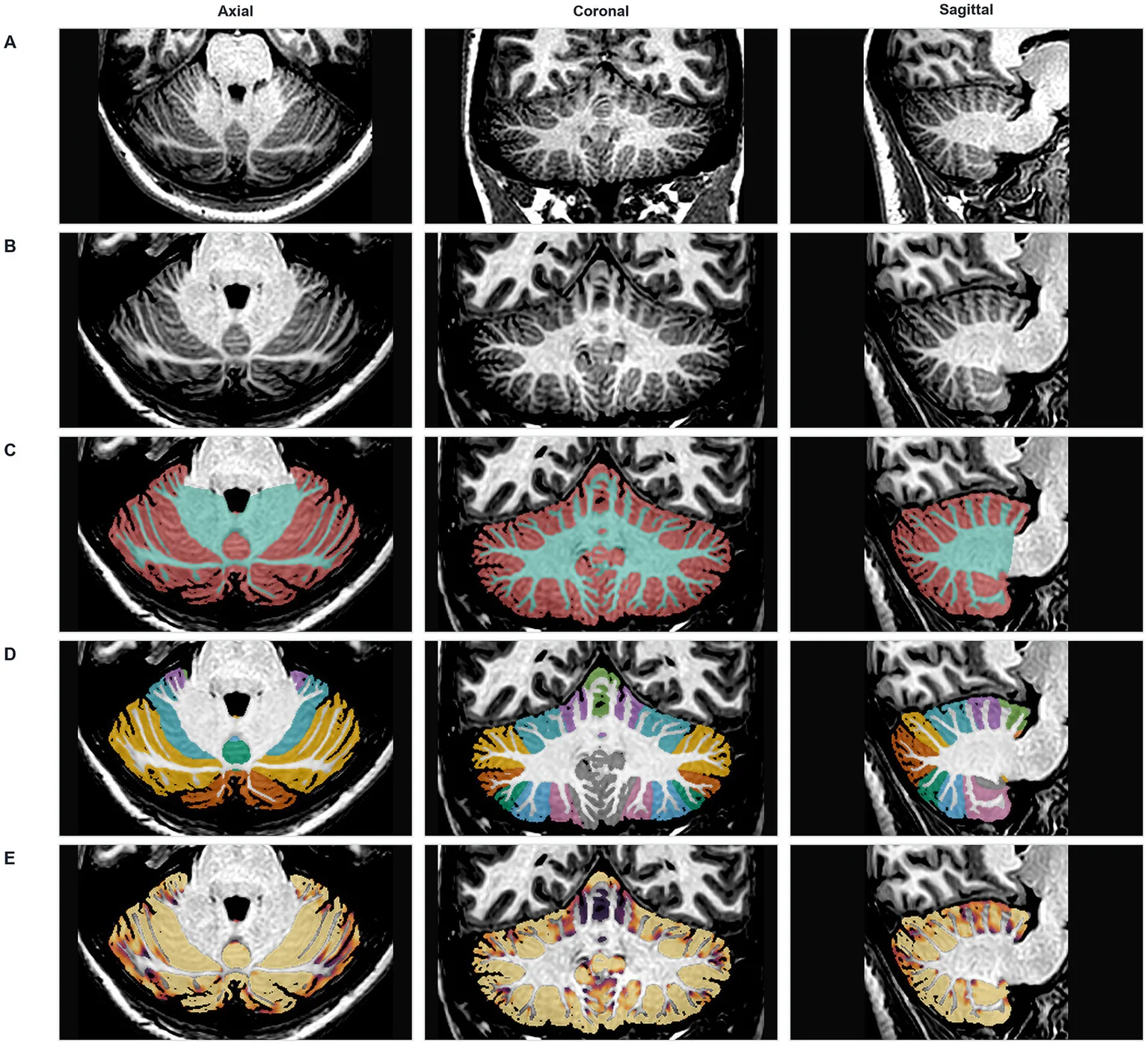
Representative cerebellar segmentation using DeepCERES. Axial, coronal, and sagittal views illustrate DeepCERES v1.1 processing in one participant. **(A)** Input T1-weighted images. **(B)** Preprocessed, super-resolved images. **(C)** Cerebellar gray matter (salmon) and white matter (turquoise) segmentation. **(D)** Cerebellar lobular parcellation. **(E)** Segmentation overlays for visual inspection. Colors in the parcellation and overlay panels distinguish anatomical labels and do not represent statistical effects. The images illustrate processing outputs rather than a quantitative assessment of segmentation accuracy.

We extracted bilateral total volume and mean thickness for each region, estimated intracranial volume (ICV), and absolute volumes of the whole cerebellum, cerebellar gray matter, and cerebellar white matter. Lobular measurements covered 12 regions: Lobules I–II, Lobule III, Lobule IV, Lobule V, Lobule VI, Crus I, Crus II, Lobule VIIb, Lobule VIIIa, Lobule VIIIb, Lobule IX, and Lobule X. Bilateral total volume was defined as the sum of left and right absolute volumes (cm^3^), and bilateral mean thickness as the arithmetic mean of left and right mean thickness values (mm). Cerebellar cortical thickness is a macroscopic estimate derived by DeepCERES from MR images and segmentation boundaries; it is not equivalent to histological cortical thickness.

### Image quality control

Two researchers independently reviewed the original T1-weighted images and DeepCERES segmentations. They assessed motion artifacts, image contrast, and the clarity of cerebellar and gray–white matter boundaries, and checked for obvious displacement of segmentation boundaries, missing labels, or labels extending beyond anatomical boundaries. Automated quality scores were also recorded. The researchers jointly reviewed any disagreements and reached a consensus. Visual quality assessment was not blinded to diagnostic group or scan time point. No participant was excluded because of image quality or an obvious segmentation error.

### Statistical analysis

All analyses were performed in R 4.6.0. Continuous variables are presented as mean and standard deviation or median and interquartile range (IQR), and categorical variables as counts and percentages. All tests were two-sided. For linear regression, *p* values and 95% confidence intervals (CIs) were calculated using heteroskedasticity-consistent (HC3) standard errors. The Benjamini–Hochberg method was used to control the false discovery rate (FDR). Adjusted *p* values are reported as q values, with *q* < 0.05 indicating significance after correction. All 95% CIs are unadjusted for multiple comparisons.

Cross-sectional volume models included group, linear and quadratic age terms, sex, and ICV. Thickness models used the same specification without ICV. We report adjusted between-group differences (TN minus HC) and their 95% CIs. Volume and thickness were treated as separate outcome families because they represent distinct morphometric measures. In both the cross-sectional and longitudinal analyses, FDR correction was applied separately to the 12 regional volume tests and the 12 regional thickness tests. Whole-cerebellum, cerebellar gray matter, and cerebellar white matter volumes were assessed in supportive analyses; these three tests formed a separate correction family within each analysis.

Clinical association analyses were restricted to patients with TN. Each model included disease duration, pain side, and Sindou grade simultaneously and adjusted for linear and quadratic age terms and sex; volume models additionally adjusted for ICV. FDR correction was applied to the 36 clinical-factor tests separately for volume and thickness. Exploratory laterality analyses used anatomical left-minus-right morphometric differences as the dependent variable and assessed the pain-side effect using the same covariate specification. The 12 volume and 12 thickness tests were corrected separately.

Longitudinal analyses used paired *t* tests. Change was defined as the postoperative minus preoperative measurement. We report mean changes, 95% CIs, and individual percentage changes. The primary paired analyses did not additionally adjust for ICV or MRI interscan interval and did not estimate rates of change.

Sensitivity analyses examined alternative age models (linear age and restricted cubic splines), additional adjustment of thickness models for ICV or automated quality scores, Wilcoxon signed-rank tests, leave-one-out analyses, and bootstrap resampling (5,000 resamples). Longitudinal sensitivity models used morphometric change as the dependent variable and included changes in ICV and automated quality scores simultaneously, with HC3 standard errors. The intercept in these models represents the estimated morphometric change when both covariate changes are zero.

We compared baseline characteristics of the 49 patients with paired MRI with those of the remaining 37 patients with TN, reporting *p* values descriptively and standardized differences for continuous variables. Postoperative clinical outcomes were summarized descriptively; associations between morphometric change and pain improvement were not tested. Each analysis included only participants with complete data for the required variables. No missing values were imputed. Full model specifications, multiple-testing families, and sensitivity analysis details are provided in the Supplementary material.

## Results

### Participant characteristics

The cross-sectional analysis included 126 participants: 86 patients with TN and 40 HCs. Mean age was 58.4 ± 12.0 years in the TN group and 56.9 ± 7.0 years in the HC group; women accounted for 60.5 and 55.0%, respectively. The TN group had a wider age distribution, spanning 30–83 years, compared with 41–75 years in HCs (Supplementary Figure S1).

Median disease duration in patients with TN was 6 years (IQR, 3–10). Right-sided pain was reported by 55 patients (64.0%), and 79 (91.9%) had purely paroxysmal pain. Among the 85 patients with medication records, 83 (97.6%) received pharmacological treatment and 72 (84.7%) had a carbamazepine-containing regimen. DeepCERES automated quality scores were lower in the TN group than in the HC group (0.916 ± 0.015 vs. 0.923 ± 0.010; *p* = 0.002; Supplementary Table S2).

Compared with the remaining 37 patients with TN, the 49 patients with paired MRI included a higher proportion of women (67.3% vs. 51.4%) and patients with left-sided pain (42.9% vs. 27.0%). Baseline Lobule V thickness was lower in the paired subgroup (standardized mean difference, −0.45; unadjusted *p* = 0.043). The comparison of the four Sindou categories recorded in the dataset yielded an unadjusted *p* value of 0.020. These comparisons were descriptive and were used to characterize the longitudinal subgroup (Supplementary Table S3).

### Cross-sectional cerebellar morphology

In the primary adjusted models, bilateral mean cortical thickness was higher in patients with TN than in HCs in Lobule IV (adjusted difference, 0.104 mm; 95% CI, 0.040–0.168; *q* = 0.020) and Lobule VI (0.071 mm; 95% CI, 0.021–0.121; *q* = 0.036). Thickness differences in the remaining 10 regions did not meet the FDR threshold. No between-group volume differences were significant after FDR correction in the 12 regions, the whole cerebellum, cerebellar gray matter, or cerebellar white matter (Figure 3; Supplementary Table S4).

**Figure 3:**
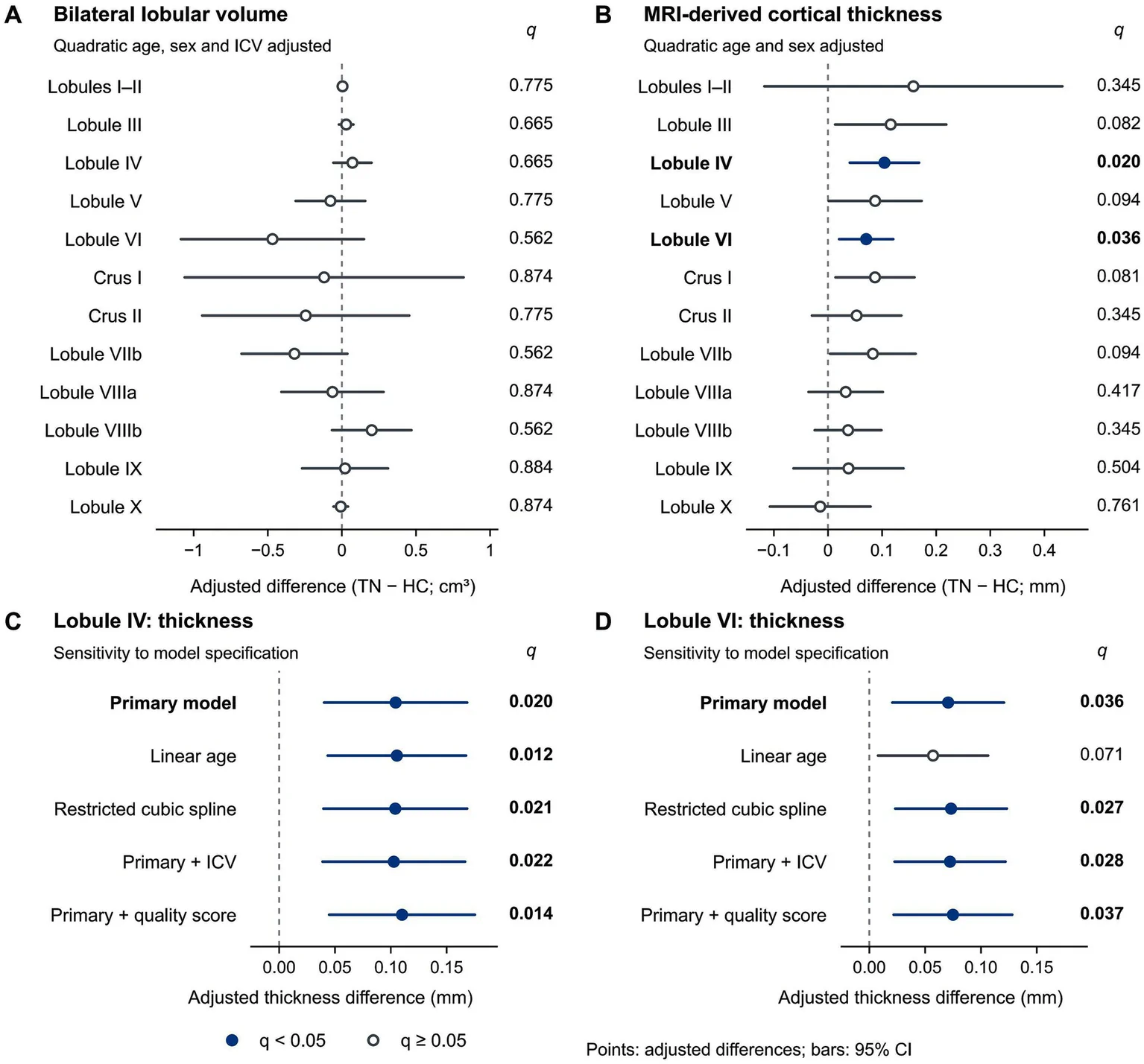
Cross-sectional cerebellar morphometry and sensitivity analyses of cortical thickness. **(A)** Adjusted differences in bilateral lobular volume and **(B)** bilateral MRI-derived mean cortical thickness between 86 patients with trigeminal neuralgia (TN) and 40 healthy controls (HCs), shown for all 12 cerebellar regions. Primary models included linear and quadratic age terms and sex; volume models additionally included intracranial volume (ICV). **(C, D)** Thickness differences in Lobules IV and VI under the primary model, alternative age models using linear age or restricted cubic splines, and primary models additionally adjusted for ICV or the DeepCERES automated quality score. ICV and quality score were added in separate sensitivity models. Points denote adjusted TN-minus-HC differences; horizontal bars show 95% confidence intervals calculated using HC3 standard errors. Dashed vertical lines indicate no difference. Blue filled circles indicate *q* < 0.05, and gray open circles indicate *q* ≥ 0.05. Confidence intervals are unadjusted for multiple comparisons. Benjamini–Hochberg false discovery rate correction was applied separately to the 12 lobular tests for each morphometric measure within each model. Panels C and D show selected results from the corresponding 12-region thickness analyses. Positive differences indicate higher measurements in TN.

### Clinical associations and laterality

Within the TN group, disease duration, pain side, and Sindou grade showed no significant associations with bilateral regional volume or mean thickness after FDR correction (Supplementary Table S5). Exploratory laterality analyses likewise found no pain-related asymmetry in volume or thickness that met the corrected significance threshold (Supplementary Table S6).

### Clinical outcomes in the paired subgroup

Of the 49 patients with paired MRI, 33 (67.3%) underwent microvascular decompression (MVD) and 16 (32.7%) underwent percutaneous intervention (PI). Postoperative pain and clinical follow-up records were available for 26 patients (53.1%). Among these patients, the median preoperative average pain score for the preceding week was 5 (IQR, 4–6), and immediate postoperative pain reduction was recorded as 100% in 21 patients (80.8%).

Time to the first clinical follow-up was recorded for 26 patients and averaged 208.3 ± 29.6 days after surgery. Pain scores at the second telephone follow-up were available for 24 patients; this assessment took place an average of 583.9 ± 99.2 days after surgery. Of these patients, 21 (87.5%) had an NRS score of 0. Second telephone follow-up pain scores were missing for 25 patients. The numbers of available and missing observations for other clinical measures are reported in Supplementary Table S7.

### Exploratory postoperative changes

Among the 49 patients with paired MRI, postoperative volume increases in Lobules IV–VI, VIIb, VIIIa, and VIIIb met the FDR threshold. Across these six regions, mean changes ranged from 0.044 to 0.103 cm^3^, and the mean of individual percentage changes ranged from 0.86 to 1.69%. Volume changes in the remaining six regions were not significant after correction.

Mean thickness increased postoperatively in Lobule IV by 0.039 mm (95% CI, 0.018–0.061; *q* = 0.003), in Lobule V by 0.066 mm (95% CI, 0.033–0.099; *q* = 0.002), and in Lobule VI by 0.037 mm (95% CI, 0.017–0.057; *q* = 0.003). The corresponding means of individual percentage changes were 1.82%, 3.00%, and 1.38%. Thickness changes in the remaining nine regions did not meet the FDR threshold.

Supportive analyses showed mean increases of 0.594 cm^3^ in whole-cerebellum volume (95% CI, 0.249–0.940) and 0.538 cm^3^ in cerebellar gray matter volume (95% CI, 0.221–0.856), with *q* = 0.002 for both. Cerebellar white matter volume change was not significant after correction (Supplementary Table S8).

### Sensitivity analyses

The cross-sectional Lobule IV thickness difference was significant after FDR correction in all three age models, with adjusted differences of 0.104–0.105 mm. For Lobule VI, the difference was 0.057 mm in the linear age model (95% CI, 0.008–0.106; *q* = 0.071) and did not meet the corrected significance threshold. The restricted cubic spline model yielded a difference of 0.073 mm (95% CI, 0.023–0.123; *q* = 0.027), close to the primary model estimate. Lobule III met the FDR threshold only in the linear age model; Crus I did not meet the threshold in any of the three models (Supplementary Table S9).

The thickness differences in Lobules IV and VI remained significant after FDR correction when ICV or automated quality scores were added to the models. Within the TN group, Lobule VI thickness was positively correlated with automated quality scores (Spearman *ρ* = 0.297; *q* = 0.022). No correlations between ICV and thickness in Lobules IV or VI met the FDR threshold in either the pooled sample or either diagnostic group (Supplementary Table S10). In cross-sectional leave-one-out analyses, adjusted thickness differences ranged from 0.093 to 0.112 mm for Lobule IV and from 0.065 to 0.077 mm for Lobule VI, with no reversal of direction. Bootstrap 95% CIs for both differences excluded zero (Supplementary Table S11).

In the longitudinal Wilcoxon signed-rank analyses, all six volume increases and all three thickness increases identified in the primary analysis met the FDR threshold. Bootstrap 95% CIs for all nine mean changes were also above zero. None of the changes reversed direction in leave-one-patient-out analyses. Volume increases in Lobules IV–VI and VIIIb and thickness increases in Lobules IV–VI were significant after correction in all 49 analyses. The volume increase in Lobule VIIb met the threshold in 23 analyses, with a maximum q value of 0.081; the increase in Lobule VIIIa met the threshold in 25 analyses, with a maximum q value of 0.073 (Supplementary Table S12).

In sensitivity models that simultaneously included changes in ICV and automated quality scores, the estimated changes at zero change in both covariates remained significant after FDR correction for volume in Lobules IV, V, and VIIIb and for thickness in Lobules IV–VI. The estimated Lobule VI thickness change was 0.028 mm (95% CI, 0.009–0.047; *q* = 0.018). The corresponding intercept tests for volume changes in Lobules VI, VIIb, and VIIIa did not meet the FDR threshold (Supplementary Table S13).

## Discussion

Our principal cross-sectional finding was higher MRI-derived mean cortical thickness in Lobules IV and VI in patients with TN, without significant regional or global cerebellar volume differences after FDR correction. The Lobule IV difference was consistent across sensitivity analyses, whereas the Lobule VI result was sensitive to how age was modeled. In the exploratory paired analysis, postoperative volume increases in Lobules IV–VI, VIIb, VIIIa, and VIIIb and thickness increases in Lobules IV–VI met the FDR threshold. Volume changes in Lobules IV, V, and VIIIb and thickness changes in Lobules IV–VI also remained significant in sensitivity models that simultaneously included changes in ICV and automated quality scores.

Previous whole-brain morphometric studies in TN have reported inconsistent cerebellar findings. Some VBM studies found lower regional cerebellar gray matter volume or density (3–5, 12), whereas another reported abnormalities in the cingulate cortex and several subcortical structures but no significant cerebellar changes (6). Shen et al. (12) observed reduced cerebellar gray matter volume in patients with a disease duration of 0–2 years, suggesting that morphometric features may differ across disease stages. Wu et al. (5) found lower right cerebellar gray matter volume in patients with TN and NVC than in HCs, but did not observe the same difference in patients without NVC. Meta-analyses have also yielded differing results. Henssen et al. (13) identified lower gray matter density in the anterior cerebellar lobe, but their analysis included both TN and trigeminal neuropathic pain and therefore could not establish a TN-specific cerebellar abnormality. Neither the meta-analysis of 13 VBM studies by Tang et al. (10) nor the activation likelihood estimation meta-analysis by Nardoni et al. (9) identified consistent cerebellar structural abnormalities across studies. VBM-derived regional gray matter density or volume and segmentation-derived absolute lobular volume are different measures and can be compared only to a limited extent. Our observation of regional thickness differences without significant volume differences adds to the structural imaging evidence in TN, which has largely relied on VBM.

The thickness differences in Lobules IV and VI, in the absence of corresponding volume differences, suggest that thickness may provide morphometric information beyond that captured by volume. Lobule IV lies in the anterior cerebellar lobe and is primarily involved in sensorimotor processing and somatosensory integration (14, 15). Research in healthy volunteers has shown that nociceptive trigeminal stimulation activates Lobule VI, Lobule VIIIa, and Crus I, and has identified functional connectivity between the cerebellum and the pons, periaqueductal gray, thalamus, and insula (16). Basedau et al. (17) further reported somatotopically organized responses in bilateral Lobules I–IV, V, VIIb, and VIIIa and Crus I following nociceptive stimulation of different trigeminal divisions and the occipital nerve. The relatively robust Lobule IV thickness difference in our study overlaps spatially with these nociception-related functional regions, providing a morphometric observation consistent with a possible role for cerebellar sensorimotor processing in TN.

Lobule VI contributes to both sensorimotor and cognitive processing. Coombes and Misra found that painful stimulation and motor tasks produced overlapping activation in Lobules VI and VIIb; these regions showed functional connectivity with the supplementary motor area, thalamus, and anterior midcingulate cortex (23). Previous functional connectivity studies and reviews have also implicated Lobules IV–VI and Crus I in pain processing (24, 25). The cross-sectional evidence for Lobule VI was less consistent than that for Lobule IV, as its difference did not meet the FDR threshold in the linear age model. Within the TN group, Lobule VI thickness was positively correlated with automated quality scores, although the group difference remained significant after adjustment for this score. Thickness differences in Lobules IV and VI appeared in both analyses, but the cross-sectional comparison assessed average differences between patients with TN and HCs, whereas the longitudinal analysis assessed within-patient change. Mean thickness in these two lobules was higher in the TN group than in HCs and increased further after surgery in the paired subgroup. This direction of change does not support interpreting the postoperative increases as normalization toward HC levels. The presence of a Lobule VI thickness difference in both the between-group comparison and within-patient follow-up makes this region of interest for further study of TN-related cerebellar morphology and postoperative change.

Previous longitudinal imaging studies in TN have focused mainly on the cerebral cortex, pain-related networks, and the trigeminal nerve itself. These studies have reported post-treatment changes in the ventral anterior insula, sensorimotor networks, and trigeminal nerve, and some have also reported reduced cerebellar functional abnormalities after treatment (26–29). In our study, postoperative changes involved several cerebellar lobules and were accompanied by increases in whole-cerebellum and cerebellar gray matter volume. In models that simultaneously included changes in ICV and automated quality scores, estimated changes at zero change in both covariates remained significant after FDR correction for volume in Lobules IV, V, and VIIIb and for thickness in Lobules IV–VI. Volume findings in Lobules VIIb and VIIIa were less stable in leave-one-patient-out analyses, indicating that robustness differed across regions. The lobules showing postoperative changes partly overlap with regions involved in trigeminal nociception and sensorimotor processing. This overlap provides a basis for further investigation of how postoperative morphometric changes relate to nociceptive input and sensorimotor modulation (16, 17).

This study has several limitations. First, it was a secondary analysis of a single-center public dataset, and participant inclusion was constrained by the available imaging and clinical data. Cross-sectional comparisons cannot establish the temporal or causal relationship between cerebellar morphometric differences and TN. Second, most patients received medication, predominantly carbamazepine. The small number of untreated patients and incomplete information on medication dose, treatment duration, and postoperative medication changes prevented us from separating medication effects from disease-related effects. Third, the longitudinal sample comprised 49 patients: 33 who underwent MVD and 16 who underwent PI. Combining procedures may obscure procedure-specific differences. Only 26 patients had postoperative pain and clinical follow-up records, limiting analyses of associations between imaging changes and clinical outcomes.

Fourth, preoperative and postoperative MRI scans were processed independently, without a subject-specific longitudinal segmentation pipeline. We had neither controls scanned repeatedly over the same period nor DeepCERES v1.1 test–retest data obtained under conditions matching those of this study. Sörös et al. (30) used an earlier CERES implementation to analyze same-day repeat MRI scans in 21 healthy participants. In the lobules corresponding to our significant findings, coefficients of variation ranged from 0.70 to 2.95% for unilateral volume and from 0.57 to 1.60% for unilateral thickness. These results offer a methodological reference for the small postoperative changes observed here, but coefficients of variation and mean changes quantify different aspects of the measurements. The software versions, scanning conditions, and definitions of unilateral and bilateral measures also differ. These data therefore cannot establish whether our observed changes exceeded measurement error. Bootstrap resampling, leave-one-out analyses, and adjustment for ICV and quality scores cannot exclude systematic measurement shifts. We also did not estimate rates of morphometric change. Thus, we cannot distinguish the contributions of surgery, pain relief, the natural disease course, and measurement variability.

Fifth, visual quality assessment was not blinded to diagnostic group or scan time point, which may have introduced observer bias. Finally, DeepCERES-derived thickness is a macroscopic morphometric estimate that depends on MR image contrast and automated segmentation boundaries. It is not equivalent to histological cortical thickness and cannot support inferences about cellular or histological changes.

## Conclusion

Patients with TN had higher MRI-derived cortical thickness in Lobules IV and VI than HCs, whereas no regional or global cerebellar volume differences were significant after FDR correction. The Lobule IV finding was consistent across sensitivity analyses; the Lobule VI finding was sensitive to how age was modeled. Paired MRI showed small postoperative increases in volume or thickness in several lobules, some of which remained significant in sensitivity models accounting for changes in ICV and automated quality scores. These postoperative findings are exploratory. Their biological significance and relationship with clinical outcomes require further assessment in longitudinal studies that include repeated MRI in controls and record clinical outcomes.

## Data Availability

The datasets presented in this study can be found in online repositories. The names of the repository/repositories and accession number(s) can be found at: https://openneuro.org/datasets/ds005713/versions/1.0.1 and https://openneuro.org/datasets/ds005713/versions/2.0.2.
