# Supplementary material for: Cerebellar lobular morphometry in trigeminal neuralgia: cross-sectional and exploratory postoperative analyses
Source: Front Neurol. 2026 Oct 22;17:1964745. doi: 10.3389/fneur.2026.1964745 (PMC13735588; doi:10.3389/fneur.2026.1964745)
Supplement: Supplementary file 1 [file Supplementary_file_1.docx]

**Figure S1. Age distributions of healthy controls (HC) and patients with trigeminal neuralgia (TN).**

Both panels use identical 5-year age bins and axis limits. Bar heights indicate the percentage of participants within each diagnostic group. Bins include their lower boundary and exclude their upper boundary.
